# Highly accelerated real-time T_2_-weighted imaging with through-time radial GRAPPA and low-latency GPU reconstruction

**DOI:** 10.1186/1532-429X-16-S1-W33

**Published:** 2014-01-16

**Authors:** Di Xu, Shuo Han, Haris Saybasili, Aravindan Kolandaivelu, Henry Halperin, Menekhem Zviman, Mark A Griswold, Nicole Seiberlich, Daniel A Herzka

**Affiliations:** 1 Biomedical Engineering, The Johns Hopkins School of Medicine, Baltimore, Maryland, USA; 2 Biomedical Engineering, Tsinghua University, Beijing, China; 3 Siemens Healthcare USA, Inc., Chicago, Illinois, USA; 4 Cardiology, The Johns Hopkins School of Medicine, Baltimore, Maryland, USA; 5 Radiology, Case Western Reserve University, Cleveland, Ohio, USA; 6 Biomedical Engineering, Case Western Reserve University, Cleveland, Ohio, USA

## Background

T_2_-weighted cardiac images are commonly used for edema detection [1-4]. However, neither black-blood TSE nor cine images can offer real-time edema monitoring, and are therefore not suitable for the guidance of cardiac ablation procedures. We proposed a radial T_2_-weighted interrupted balanced SSFP (rT_2_W-iSSFP), a real-time high temporal resolution sequence targeted at monitoring edema.

## Methods

### Sequence

**Figure 1:**
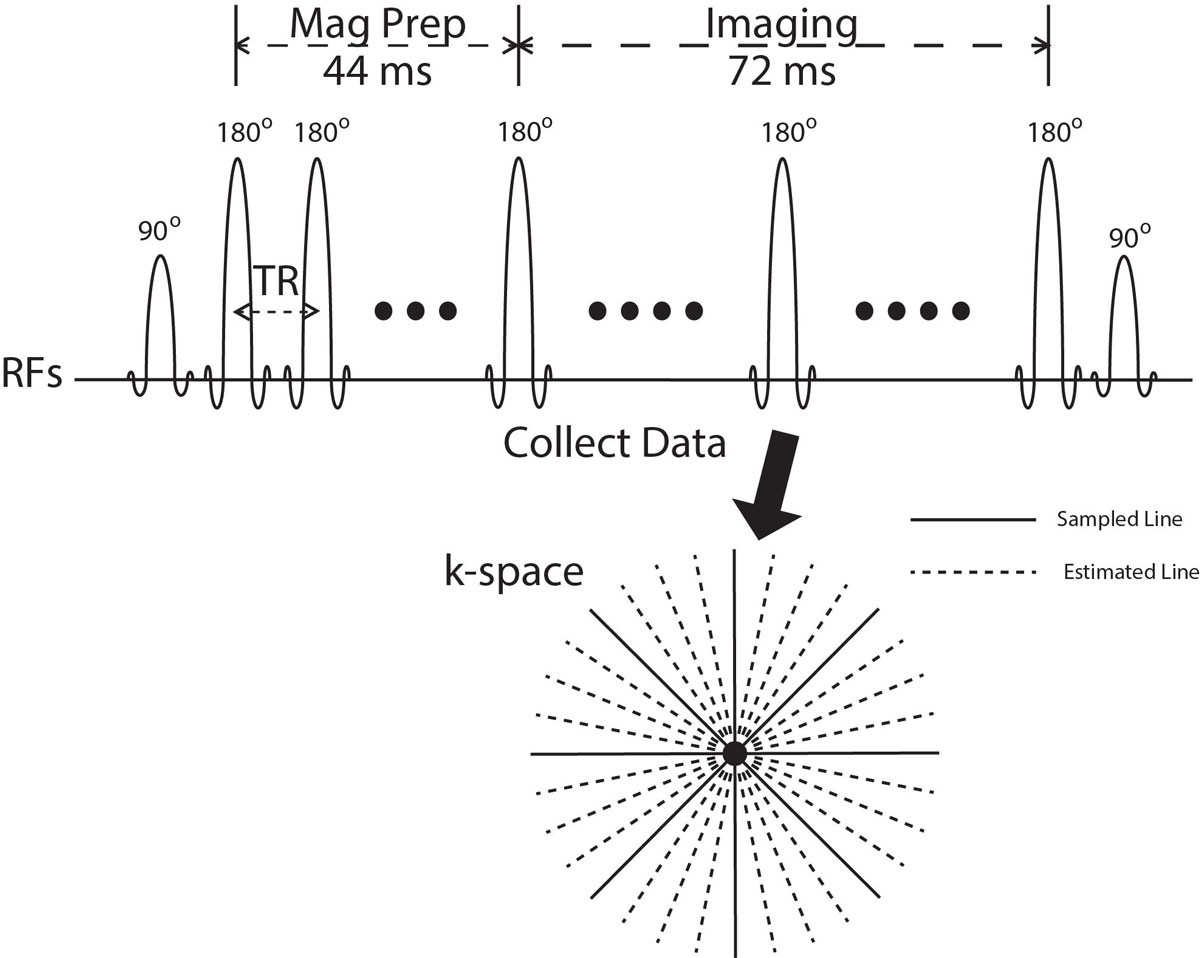
**Illustration of the acquisition scheme, flip angles and k-space sampling pattern of rT_2_W-iSSFP**. Four-fold acceleration is shown in this example. Images were reconstructed using through-time radial GRAPPA with a low-latency implementation. Typically a 192 × 192 matrix and an acceleration rate of R = 8 is used.

rT_2_W-iSSFP generates T_2_-weighting with a series of 180° RF pulses. TE-effective for the radial sequence is defined as the time from the beginning of the train to the median imaging echo. rT_2_W-iSSFP also incorporates through-time radial GRAPPA to achieve high temporal resolution with high degrees of acceleration (R = 8) [5], (Figure 1) which was implemented on a 48-core hybrid system with a GPU (Tesla C1060, NVIDIA), achieving 10-20 fps image acquisition with 20 ms latency reconstruction and image display [6].

### Simulations

**Figure 2:**
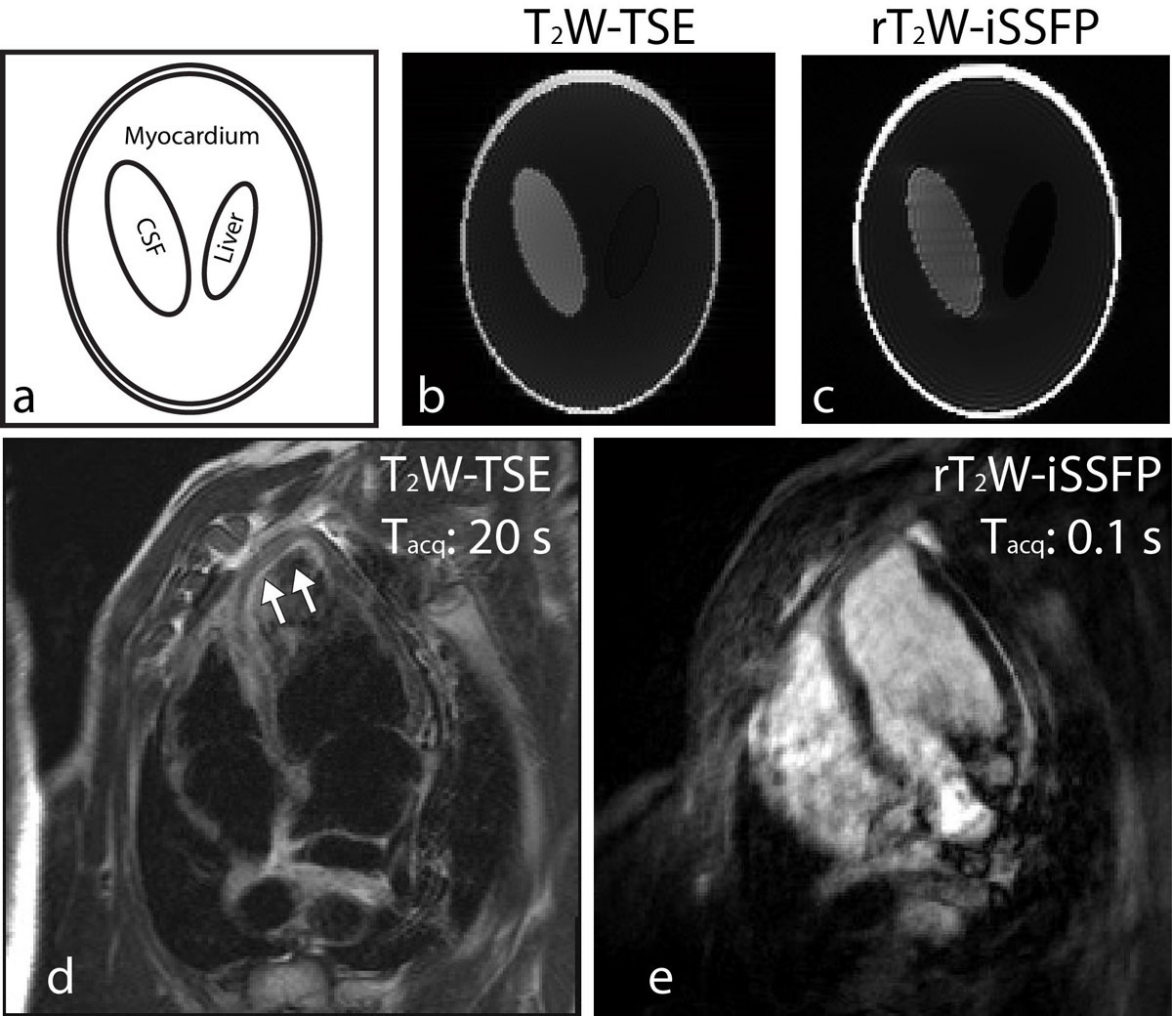
**(a) Illustration of the Shepp-Logan phantom used for simulation**. (b) T_2_W-TSE and (c) r T_2_W-iSSFP were simulated. Swine with acute injury was scanned by (d) breath-hold T_2_W-TSE and (e) free-breathing r T_2_W-iSSFP as well. The edema (the arrows in d) is depicted in both d and e. In both simulation and in-vivo imaging, T_2_W-TSE was used as the reference of T_2_-weighting. T_acq _- acquisition time.

Bloch equation simulations were performed to evaluate the T_2_-weighting and the image quality of rT_2_W-iSSFP using a variant of the Shepp-Logan phantom containing 3 ellipsoids with different T_1_s and T_2_s to represent cerebrospinal fluid (CSF), liver, and myocardium (Figure 2a) [7,8]. T_2_-weighted turbo spin echo (T_2_W-TSE) was also simulated [3,9]. TE-effective was 60 ms for both T_2_W-TSE and rT_2_W-iSSFP.

### Animal Model

Swine with acute injury (*N = 2*) were imaged on a 1.5T scanner (Avanto, Siemens, Germany). Free-breathing ECG-triggered single-shot rT_2_W-iSSFP was acquired (TE-effective = 80 ms; TR = 3 ms; matrix = 192 × 192; 3 slices per heartbeats). ECG-triggered, breath-held T_2_W-TSE (TE = 80 ms, resolution = 1 × 1 mm^2^, matrix = 192 × 192) was used as a reference.

## Results

The results from simulation of T_2_W-TSE and rT_2_W-iSSFP are shown in Figures 2b and 2c. The intensity difference between CSF and liver is similar in T_2_W-TSE and rT_2_W-iSSFP. Streaking artifacts are seen in Figure 2c, but these are not pronounced in in vivo images. Four-chamber views of swine heart from T_2_W-TSE (breath-hold) and rT_2_W-iSSFP (free-breathing) are shown in Figures 2d and 2e. Edema at the anteroseptal region due to acute myocardial infarction is depicted (the arrows in d).

## Conclusions

rT_2_W-iSSFP offers high temporal resolution T_2_-weighted imaging with image quality sufficient for visualization of edema from acute injury. rT_2_W-iSSFP can be applied to real-time monitoring of edema formation during cardiac interventions.
